# Reconfigurable Orbital Electrowetting for Controllable Droplet Transport on Slippery Surfaces

**DOI:** 10.3390/mi16060618

**Published:** 2025-05-25

**Authors:** Jiayao Wu, Huafei Li, Yifan Zhou, Ge Gao, Teng Zhou, Ziyu Wang, Huai Zheng

**Affiliations:** 1 The Institute of Technological Sciences, Wuhan University, Wuhan 430072, China; 2 School of Power and Mechanical Engineering, Wuhan University, Wuhan 430072, China; 3 Mechanical and Electrical Engineering College, Hainan University, Haikou 570228, China

**Keywords:** reconfigurable droplet manipulation, orbital electrowetting, controllable transport, surface charge, slippery surfaces

## Abstract

The controllable transport of droplets on solid surfaces is crucial for many applications, from water harvesting to bio-analysis. Herein, we propose a novel droplet transport controlling method, reconfigurable orbital electrowetting (ROEW) on inclined slippery liquid-infused porous surfaces (SLIPS), which enables controllable transport and dynamic handling of droplets by non-contact reconfiguration of orbital electrodes. The flexible reconfigurability is attributed to the non-contact wettability modulation and reversibly deformable flexible electrodes. ROEW graphically customizes stable wettability pathways by real-time and non-contact printing of charge-orbit patterns on SLIPS to support the continuous transport of droplets. Benefiting from the fast erase-writability of charges and the movability of non-contact electrodes, ROEW enables reconfiguration of the wetting pathways by designing electrode shapes and dynamically switching electrode configurations, achieving controllable transport of various pathways and dynamic handling of droplet sorting and mixing. ROEW provides a new approach for reconfigurable, electrode-free arrays and reusable microfluidics.

## 1. Introduction

The controllable transport of droplets on solid surfaces is crucial for many applications, from water harvesting to bio-analysis [1,2,3,4,5,6,7]. A conventional platform to achieve continuous and controllable droplet transport is microchannel-based microfluidics [8]. However, its predefined and fixed channels lead to a lack of reconfigurability, which fails to meet the requirement for on-demand regulation and dynamic decision-making of droplet motion [9,10,11,12].

Recently, emerging programmable external stimuli, including optical [13,14,15,16], magnetic [17,18], acoustical [19,20], mechanical [21], and electrical stimuli [22,23,24,25,26,27], have been introduced into droplet manipulation to improve its flexible reconfigurability. Among them, digital microfluidics based on conventional electrowetting (CEW) has been noticed and applied due to its high programmability, maneuverability, and spatial-temporal precision, and is expected to replace the traditional fixed-operation microchannel-based microfluidics [28,29,30]. Despite significant progress, CEW is limited by the ability of solid-liquid interfacial energy regulation, leading to complex stepwise strategies for droplet manipulation. Therefore, single droplet directional transport requires a series of electrodes and a high level of synchronization between electrode activation and droplet response [30,31]. It poses a complex multi-electrode cooperative control problem in high-throughput droplet transport [32]. Although orbital electrowetting (OEW) for droplet manipulation on superhydrophobic or slippery surfaces has recently been developed by introducing orbital electrodes to replace the complex electrode arrays in CEW, it still lacks reconfigurability due to the reliance on predefined and fixed contact electrodes [29,33]. Therefore, there is an urgent need to develop droplet microfluidics that can be flexibly programmed and dynamically reconfigured without complex electrode arrays.

Here, building on our previous works for droplet manipulation by non-contact printing of charge patterns [34], we further propose the reconfigurable orbital electrowetting (ROEW), which enables controllable transport of droplets on inclined slippery liquid-infused porous surfaces (SLIPS). The reconfigurability of ROEW benefits from the wettability modulation mechanism of the non-contact printed charge-orbit patterns and the reversibly deformable flexible electrodes. The electrode pairs generate corona discharges and non-contact directional deposition of positive and negative charges on the SLIPS. Due to the asymmetric design of the electrode pairs, positive and negative charges are deposited asymmetrically on the SLIPS, forming a negatively charged orbit pattern. The negatively charged orbit pattern can graphically create wettability pathways. The real-time deposition of charge enables the wettability pathways to be retained durably, thus supporting the continuous transport of droplets.

Combined with the reusable flexible electrodes, ROEW enables continuous droplet transport along various pathways by activating a single electrode, simplifying the stepwise droplet driving strategy in CEW. Different from OEW with fixed contact electrodes, ROEW can meet the demand for dynamically reconfigurable handling of droplet sorting and mixing by switching the orbital electrode configuration, due to the fast erase-writability of charges and the movability of the non-contact electrodes. Significantly, ROEW facilitates controllable droplet transport and dynamically reconfigurable droplet handling by on-demand deformation and real-time switching of electrodes, providing a promising method for reconfigurable, electrode-free arrays and reusable digital microfluidics.

## 2. Materials and Methods

### 2.1. Materials

The material of the microneedle electrodes is steel needles (tip diameter 54 μm), purchased from Besee Ltd., Tianjin, China. The material of the flexible electrodes is conductive copper foil (thickness 20 µm), purchased from Penyida Technology Ltd., Shenzhen, China. The material of the porous substrate is polypropylene filter film (thickness 150 μm, pore size 0.8 μm), purchased from Haining Lianzhong Filtration Equipment Technology Ltd., Haining, China. The material of the droplet is DI water, purchased from Guangzhou Hewei Pharmaceutical Technology Ltd., Guangzhou, China. The food dye was purchased from Century Jomei Import and Export Trading Ltd., Tieling, China. The silicone oil (PMX-200 100 cSt) was purchased from Sinopharm Chemical Reagent Ltd., Shanghai, China.

### 2.2. Methods

#### 2.2.1. Design of the Experimental Setup

All experiments were performed on the self-built experimental setup of Figure S1. The experimental setup consists of microneedle electrodes, SLIPS, flexible bladed electrodes, an inclinable platform, a high-voltage DC power supply, and other auxiliary measuring instruments. The fabrication processes of the microneedle electrodes and the bladed electrodes were shown in Figures S2 and S3, respectively. As shown in Figure S4, a magnet-controlled fixation device was designed to hold the bladed electrodes, which were made from acrylic hardboard cut by a CO_2_ laser engraver (Julong Laser Equipment Ltd., Liaocheng, China) and embedded with magnets. The bladed electrode was placed in the slot of the fixation device. Two pairs of magnets embedded in the side plates are used to tighten the blade electrode and prevent it from moving, while the magnets embedded in the base are used for magnet-controlled movement and positioning that supports flexible blade electrodes to deform as desired.

A leveling instrument was used to ensure that the three planes where the microneedle electrodes, SLIPS and blade electrodes were located were parallel to each other. An opto-mechanical platform was used to ensure that the heights of h_1_ and h_2_ were maintained between the SLIPS and the microneedle electrodes and the bladed electrodes, respectively, across the whole area. An inclinable platform was used to adjust the incline angle of the experimental setup on demand. A high-voltage DC power supply (Dongwen High Voltage Power Supply Ltd., Tianjin, China) was used to supply the electrodes with voltages from 0 kV to 30 kV. A syringe pump (Model LSP01-1B, Ditron Electronic Technology Ltd., Baoding, China) was used to generate a continuous droplet flow.

#### 2.2.2. Preparation of Slippery Surfaces

To reduce friction, the porous substrates were soaked in a pool of silicone oil for 10 min to inject the silicone oil into the pores. Finally, the porous substrates were removed and emptied of excess silicone oil, thus preparing slippery liquid-infused porous surfaces.

#### 2.2.3. Preparation of Electrodes

For the preparation of the microneedle electrodes, we first used a high-precision CO_2_ laser engraver to cut uniform size arrays of holes in acrylic hardboard to be used as a substrate for supporting and fixing the needle electrodes (Figure S2a). Then, we embedded the needle electrodes of uniform length into the hole arrays and ensured that the spacing and height of the electrodes were consistent (Figure S2a). Finally, we connected the needle electrodes in series with copper wires to make them conductive to each other, thus preparing needle array electrodes with consistent spacing and height (Figure S2b). To enable large area uniform charge deposition on the slippery surfaces, we experimentally optimized the spacing between the needle electrodes in the needle arrays to be 20 mm (Figure S2b).

For the preparation of the flexible blade electrodes, we selected reversibly deformable thin copper foil as the electrode material and referred to the stamping and pressing process of metal plates to sharpen the top side of the copper foil electrode (Figure S3). First, we placed a thin copper foil on a smooth and flat surface. Then, we used a scraper to repeatedly scrape the copper foil directionally to eliminate the burrs on the edges of the copper foil until the edges of the copper foil were sharp. Finally, we verified that the prepared copper foil electrodes were qualified by loading the prepared copper foil electrode with a high voltage and measuring whether the tips were corona discharged with an electrostatic meter.

#### 2.2.4. Electrical Measurements

The charge quantity of the droplets was measured by a charge tester (Model 111A, Huace Test Instruments Co., Ltd., Beijing, China). The surface current density was measured by a self-built device, which consisted of an electrostatic meter (Model 6517B, Keithley Instruments, Co., Ltd., Beaverton, OH, USA), a digital multimeter (Model M6500, Keithley Instruments, Co., Ltd., Beaverton, OH, USA), and matching program-controlled software.

#### 2.2.5. Droplet Behavior Measurement

A contact angle meter (Model DSA25B, KRÜSS Scientific Instruments, Co., Ltd., Hamburg, Germany) was used to measure the contact angle of the droplets. To observe the droplet behavior, the droplets were color-marked with food dyes. A high-definition video camera (Model A7M3, Sony, Co., Ltd., Tokyo, Japan) was used to record droplet motion. Tracker software version 6.3.0 was used to extract the trajectories of the droplets.

## 3. Results and Discussion

### 3.1. Conceptual Design of ROEW on Inclined SLIPS

**Figure 1:**
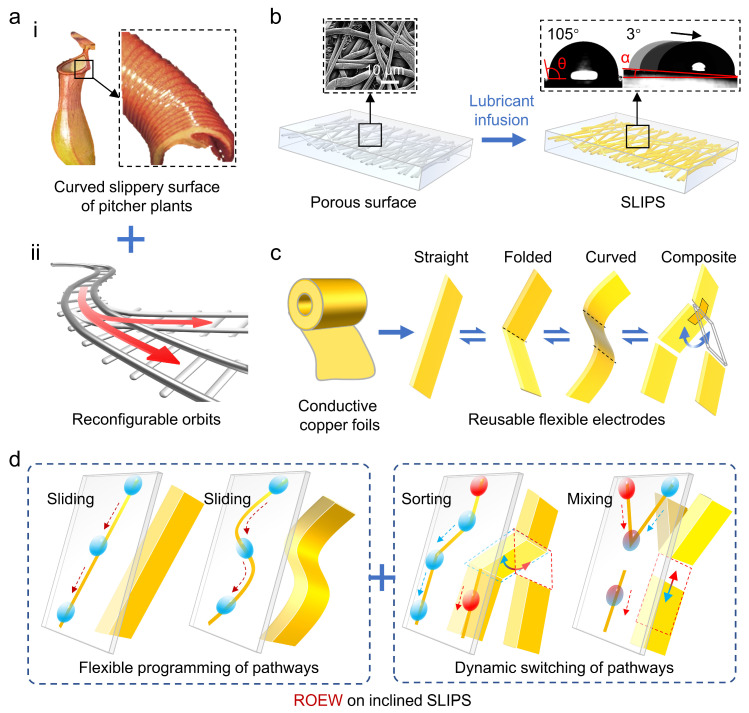
Prototype of reconfigurable orbital electrowetting (ROEW) on inclined slippery liquid-infused porous surfaces (SLIPS). (**a**) Design inspiration of SLIPS [35] (i) and ROEW (ii). (**b**) Preparation and characterization of SLIPS. The insets show the SEM characterization of the porous substrate, the hydrophobicity of the SLIPS, and the superlubricity characterization, respectively. (**c**) Design of reusable flexible electrodes and realization of cyclic deformation of straight, folded, curved, and composite-shaped electrodes. (**d**) Demonstration of ROEW for versatile droplet manipulation on inclined SLIPS by dynamically reconfiguring non-contact electrodes.

Inspired by pitcher plants and Legos, we propose the concept of reconfigurable orbital electrowetting (ROEW) for controllable transport of droplets on inclined slippery surfaces. Recently, various functional surfaces with special wettability properties for the directional transport of droplets have been developed by mimicking natural constructions [35,36,37,38,39,40,41]. To realize the low-resistance mobility of droplets, we designed slippery liquid-infused porous surfaces (SLIPS) that outperformed their natural counterparts [14] and have low adhesion properties (sliding angle: ~3°), inspired by the curved slippery surface of pitcher plants (Figure 1a(i)). SLIPS provide hydrophobic (contact angle: ~105°) and anti-fouling slippery surfaces for fast and loss-free transport of droplets (Figure 1b).

For controllable transport of droplets on SLIPS, we were inspired by Lego-like reconfigurable orbits (Figure 1a(ii)) and designed the underlying electrode arrays in conventional electrowetting (CEW) as orbital electrodes, which were not contact placed under SLIPS. To realize the reversible deformation of a single orbital electrode among various shapes, we selected lightweight, thin, and flexible conductive copper foils as the orbital electrode material (Figure 1c). The reversibly deformable and flexible material supports the electrodes to deform into various shapes, including straight, folded, and curved shapes. Therefore, the electrodes are reusable and reconfigurable, enabling flexible adaptation to applications with different droplet transport pathways. Furthermore, by customizing combinations of single-shape electrodes to construct composite electrodes with integrated switches, dynamic handling of controlled transport of droplets can also be realized.

Different from existing microfluidics that lack reconfigurability due to fixed contact electrodes or channels, ROEW is reconfigurable because the electrodes are non-contact and movable. The reconfigurability of ROEW is attributed to the droplet manipulation mechanism adopted for non-contact printing of charge patterns to regulate wettability [34]. Its reconfigurability is reflected in the flexible programming of electrode shapes and dynamic switching of electrode positions, resulting in multifunctional droplet manipulation (Figure 1d). For example, ROEW realizes the directional transport of droplets in various pathways on inclined SLIPS by non-contact flexible programming of the electrode shapes. Furthermore, it enables dynamic handling in droplet transport, such as sorting and mixing, by dynamically controlling switches in multi-pathways composite electrodes.

### 3.2. Construction of ROEW and Controllable Droplet Sliding

**Figure 2:**
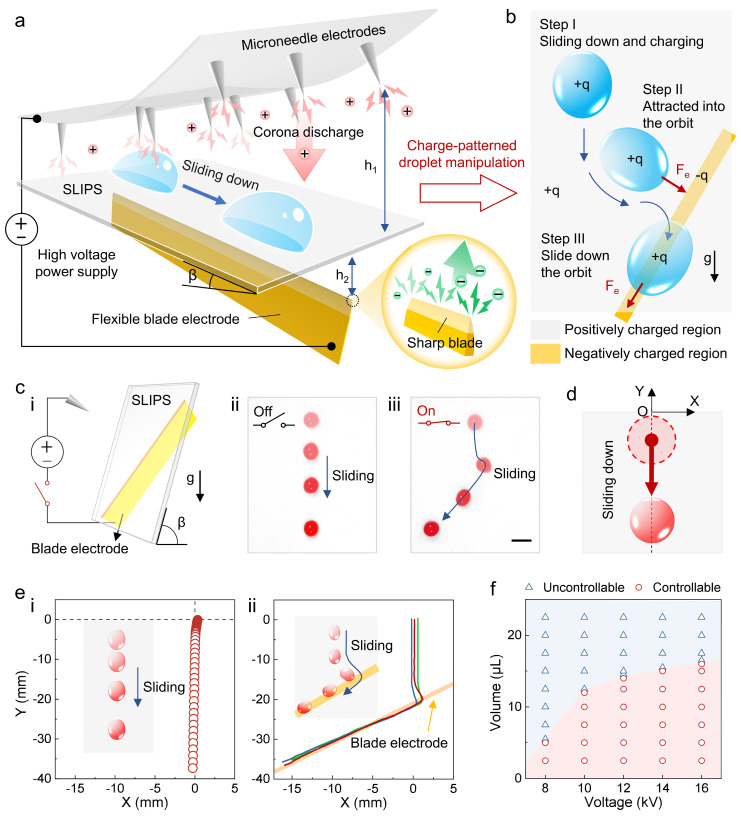
Construction of ROEW and controlled droplet sliding. (**a**) Schematic diagram of the experimental setup. Asymmetrically designed electrode pairs introduce corona discharges under high voltage to enable non-contact printing of charge-orbit patterns on SLIPS. h_1_ = 60 mm, h_2_ = 5 mm. (**b**) Schematic diagram of the mechanism of charge-orbit patterns controlling droplet transport trajectories by inducing electrostatic attraction. (**c**) Sliding behavior of droplets on inclined SLIPS. (i) is a schematic diagram of the experimental setup. (ii,iii) are sequential images of the droplet sliding in the natural case and assisted by the charge-orbit pattern (12 kV), respectively. The curves of different colors represent data from different droplet sliding trajectories. (**d**) Schematic diagram of the coordinate system for the droplet motion. (**e**) Sliding trajectories of droplets in the natural case (i) and assisted by charge-orbit patterns (ii). (**f**) Controllability of droplet volume and applied voltage on droplet orbital sliding. The scale bar is 5 mm.

Based on the conceptual design and vision of ROEW, we built the ROEW system and realized the controlled sliding of droplets. The experimental setup of ROEW consists of a microneedle electrode array, a blade electrode, SLIPS, and a high-voltage DC power supply (Figure 2a and Figure S1). The distances between the positive and negative electrodes from the SLIPS are h_1_ and h_2_, respectively (h_1_ is much larger than h_2_). The inclination angle of the SLIPS is β. The high-voltage DC power supply is used to provide the high voltage required for corona discharge to the positive and negative electrodes. As charge sources, the top positive microneedle electrode array and the underlying negative blade electrode were used to generate positive and negative charges, respectively, by corona discharge under high voltage. The charges are deposited non-contact on the SLIPS under the action of the electric field.

The mechanism of the charge-orbit patterns that drive the directional motion of the droplets is schematically shown in Figure 2b. Due to the asymmetric design of the electrode pairs, positive and negative charges are deposited asymmetrically on the SLIPS and form charge-orbit patterns. The region above the straight blade electrode deposits a negatively charged orbit while the remaining region deposits positive charges. A droplet naturally slides down on the inclined SLIPS assisted by gravity and is positively charged under corona discharge. When approaching the negatively charged orbit, it is electrostatically attracted into the orbit and slides down the orbit, thus realizing the directional transport of the droplets on the inclined SLIPS.

To verify the feasibility of ROEW, we compared the dynamic evolution of droplet sliding on inclined SLIPS in the natural case (Figure 2c(ii)) and in the underlying straight-blade electrode configuration (Figure 2c(iii)), respectively. Different from the vertical sliding of the droplet in the natural case, the sliding pathway of the droplet under the action of ROEW is altered (Supplementary Movie S1). Moreover, the contact angles of the droplet in advance and rear in the direction of motion under the action of ROEW are reduced compared with the natural sliding of the droplet (Figure S5). To investigate the sliding behavior of droplets, we established a coordinate system with the initial position of droplets as the origin and analyzed their trajectories (Figure 2d). The sliding trajectory of the droplet in the natural case is almost straight (Figure 2e(i)), while the trajectory under the action of ROEW is significantly curved (Figure 2e(ii)). Initially, the droplet naturally slides vertically. When approaching the underlying blade electrode, the droplet turns and is deflected toward the underlying electrode until it reaches the top of the underlying electrode. Then, the droplet slides along the underlying electrode until it detaches from the SLIPS. After repeated experiments, it is found that the sliding trajectories of droplets under the action of ROEW coincide with the underlying electrode. This indicates that ROEW enables orbital-controlled transport of droplets and promises to program the transport pathways by designing electrodes. To realize controllable droplet transport, we investigated the effects of droplet volume and applied voltage on the droplet orbital sliding. Figure 2f shows the critical volume and critical voltage that enables stable orbital sliding of droplets. The controllable sliding of the droplet depends on the electrostatic attraction force on the droplet and the component force of gravity on inclined SLIPS. When the former dominates, the droplet slides down the orbit (controllable slide), while when the latter dominates, the electrostatic force is not enough to support the droplet to slide down the orbit (uncontrollable slide). Therefore, we can control the droplet volume and regulate the applied voltage to realize the controllable slide of the droplet. Furthermore, the maximum velocity of the droplet sliding can reach 75 mm/s. Droplet types are universal; HCl, NaCl, NaOH solutions of different pH values, and even ethanol with low surface tension and colorant with high viscosity can be well manipulated by ROEW (Figure S6 and Supplementary Movie S2). To verify the stability of ROEW, we conducted more than 100 experiments for each type of droplet manipulation and several reduplicate experiments on the same SLIPS. Moreover, previous work has performed up to 300 repetitive droplet manipulation experiments on a similar experimental setup [34]. The numerous stable experimental results and the persistence of SLIPS are sufficient to demonstrate the stability of ROEW.

### 3.3. Mechanism Validation and Reconfigurability of ROEW

**Figure 3:**
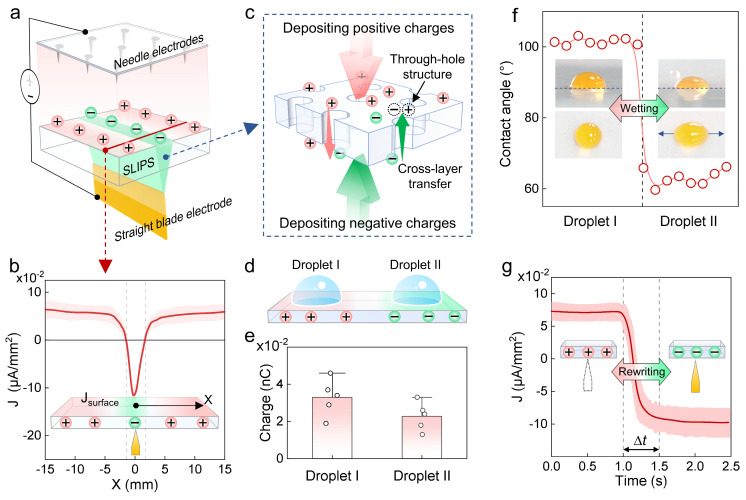
Mechanism validation and reconfigurability of REOW. (**a**) Schematic of non-contact printing of charge-orbit patterns. (**b**) Measurement of surface current density in the red region in the inset of Figure 3a. (**c**) Schematic of the through-hole structure of SLIPS to enable charge cross-layer transfer. (**d**) Droplets placed on the positive charge deposition region and negative charge orbit on the SLIPS were used for experimental verification of the mechanism. (**e**) Measurement of the charge quantity of droplets. (**f**) Measurement of droplet contact angle. The insets show the change in wettability of the droplet and the deformation of the droplet along the electrode direction, respectively. (**g**) Variation of surface current density on SLIPS before and after removal of the underlying electrode.

To realize flexible and reconfigurable droplet manipulation, we further validated the droplet manipulation mechanism of ROEW by experimental measurements and theoretical analyses, then explored its reconfigurable realization routes. To visualize the charge-orbit pattern, we modeled the charge deposition of the experimental setup of ROEW (Figure 3a) and online-measured the surface current density in the red region on SLIPS according to Warburg’s theory [42]. Figure 3b shows that the charge distribution is characterized by an orbital pattern, with the negative charge deposition region located above the electrode and the positive charge deposition region located in other surrounding regions. The result proves that the through-hole structure in SLIPS allows cross-layer charge transfer (Figure 3c). Due to the asymmetric design of the spacing and shape of the electrode pairs, positive and negative charges are deposited asymmetrically on the SLIPS. The through-hole structure of SLIPS promotes cross-layer charge transfer and dynamic neutralization, resulting in the formation of an orbital pattern consisting of positive and negative charges (Figure 3a).

To verify the mechanism by which charge-orbit patterns manipulate droplets by modulating wettability, we measured the charge quantity, contact angle, and deformation of droplets (Figure 3d). The droplets are positively charged on SLIPS with charge-orbit patterns (Figure 3e). Owing to the charge distribution on SLIPS, different regions show significant differences in wettability (Figure 3f). The contact angle of droplets on negative charge orbits (~60°) is lower than that of the surrounding positive-charge region (~90°). Further observation reveals that the droplet deformation on negative charge orbits is obvious. The droplets are stretched from a round shape to an oval shape along the direction of the underlying blade electrode. These further confirm the mechanism of controlled transport of droplets (Figure 2b). The negatively charged orbits achieve graphical modulation of wettability by inducing the generation of electrostatic forces. Under this regulation mechanism, positively charged droplets are attracted into the orbit, and wetting pathways are established, supporting controllable droplet transport. Furthermore, the charge-orbit patterns have self-healing properties due to the real-time charge replenishment, which allows the wetting pathways to be stably established to support the continuous transport of droplets.

To investigate the dynamic reconfigurability of ROEW, we online-measured the dynamic evolution of the surface current density before and after the switching of the underlying blade electrode. As shown in Figure 3g, when the underlying electrode is activated, the region above the electrode presents a stable negative charge distribution. After the electrode is removed, the charge polarity is significantly reversed and the surface current density jumps from negative to positive within 0.5 s. This polarity reversal indicates that the non-contact and real-time charges deposited have fast erase-writability. Due to the removal of the underlying electrode (source of negative charge), the original negative charge is rapidly neutralized and redeposited with positive charges from the positive real-time deposition above.

Due to the fast erase-writability of charges and the reversible deformation and non-contact movability of electrodes, there are two ways to reconfigure the charge-orbit patterns by changing the shape and position of electrodes. Attractively, they achieve distinctive droplet manipulation effects. For example, by programming the shape of a single electrode, the design of various droplet transport pathways, including straight lines, folded lines, and curved lines, can be realized. Furthermore, by constructing locally movable multi-pathway electrodes and dynamically switching the local positions of the electrodes to refresh the local charge, dynamic switching of droplet transport pathways can be realized.

### 3.4. Flexibly Programmable Droplet Transport

**Figure 4:**
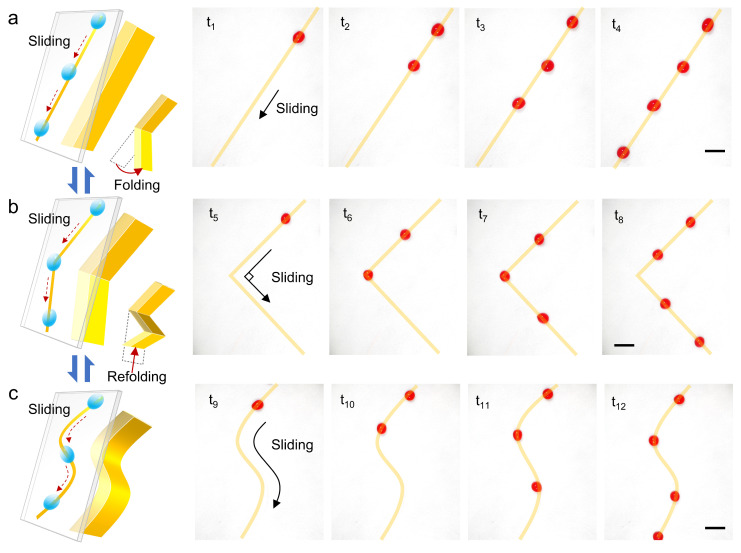
Continuous transport of droplets in various complex pathways is achieved by flexible programming of reusable electrode shapes. (**a**) Straight pathway. (**b**) Folded 90° pathway. (**c**) S-shaped pathway with double bends. The scale bar is 5 mm.

Recent advances in droplet-based microfluidics have highlighted the demand for the ability to programmatically control the electrode shape or surface topography to provide various droplet transport pathways for flexible adaptation to diverse applications [43,44,45]. ROEW enables self-repairing and persistent retention of charge-orbit patterns through real-time charge printing, thereby establishing a stable wetting channel to support continuous droplet transport. As shown in Figure 4a and Supplementary Movie S3, continuous and stable transport of droplets along a straight pathway on inclined SLIPS is achieved by using a simple straight blade electrode.

The flexible programming of droplet transport pathways is the key to realizing the self-configuration of ROEW for various application scenarios. Benefiting from the reversible deformation of the flexible electrodes, the flexible programming of various complex pathways for droplet transport is realized by cyclically deforming the shape of the electrodes to reconfigure the wettability pattern. For example, the switching of the droplet transport pathway from straight to V-shape was realized by folding one-half of the straight flexible electrode (Figure 4b and Supplementary Movie S3). Further, the switching of the droplet transport pathway from V-shaped to S-shaped was realized by folding one-fourth of the V-shaped electrode (Figure 4c and Supplementary Movie S3). These results show that REOW is not only able to flexibly adapt to complex pathways such as a 90° sharp turn and continuous bends in an S-shape but also verifies the feasibility of flexible reconfiguration of the droplet transport pathways by programming the electrode shapes.

Notably, by designing the shape of the flexible electrodes, ROEW constructs the basic functional units for directional droplet transport in straight and curved lines and controllable droplet steering in right-angle bends and curved bends. These droplet transport and steering functional units can be assembled like a jigsaw puzzle or Lego to build a reconfigurable fluid network to meet the specific needs of the user, which enhances the flexible adaptability of ROEW to various application scenarios. Furthermore, the reversible deformation of the flexible electrodes makes a single electrode reusable. By cyclically deforming the geometrical configuration of a single electrode, ROEW enables flexible switching of various droplet transport pathways on the same SLIPS, which is of great significance for programmable and reconfigurable droplet microfluidics without electrode arrays.

### 3.5. Dynamically Reconfigurable Droplet Handling

Dynamically reconfigurable droplet microfluidics for on-demand droplet handling has emerged as a common technological requirement for enabling efficient bioassay and spatiotemporally controlled chemical analysis [46,47,48,49]. Based on programming the shape of a single flexible electrode to realize the continuous transport of droplets, we constructed composite electrodes by integrating dynamic switches in the transport pathways, thereby realizing dynamic handling and real-time decision-making for continuous droplet flow. The dynamic reconfigurability of ROEW is attributed to the fast erase-rewritable property of the charge-orbit patterns and the flexible movability of the non-contact electrodes. By on-demand control of the conduction and disconnection of the switches in the composite electrodes, designated branches in the droplet transport network can be selectively activated, enabling dynamic reconfiguration of droplet handling in a continuous droplet flow.

**Figure 5:**
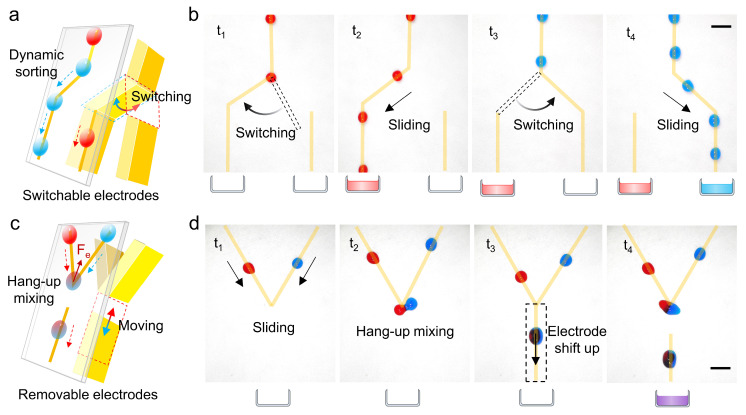
Dynamic handling of droplets is realized by controlling switches in the composite electrodes. (**a**) Schematic diagram of a composite electrode with a Y-shaped fork switch for droplet sorting. (**b**) Sequential images of droplet sorting are realized by dynamically switching the switches to selectively activate the pathway. (**c**) Schematic diagram of a V-shaped composite electrode with a movable pin switch for droplet mixing. (**d**) Sequential images of droplet temporal mixing and transport are achieved by dynamically moving the pin switch to control its conduction or disconnection from the V-shaped electrodes. The scale bar is 5 mm.

To demonstrate the dynamic droplet handling capability of ROEW, we designed two composite electrodes with representative switches to realize online sorting and temporal mixing of droplets. Since droplet sorting is characterized by a single input and multiple outputs, we designed a composite electrode with a Y-shaped fork switch (Figure 5a). By recognizing the droplet signals and dynamically switching the electrodes, the online sorting of droplets with different characteristics is realized. As a demonstration, we performed dynamic sorting of droplets of different colors (Supplementary Movie S4). As shown in Figure 5b, the droplet flow slides directionally along a straight pathway. When the red droplets flow through the detection area, the flexible electrode switch is switched to the left side, activating the left pathway to sort the red droplets into the left collection cell. Conversely, the blue droplets are sorted into the right collection cell as the switch is switched to the right side, thus realizing online sorting of different colored droplets.

Whereas droplet mixing is characterized by multiple inputs and a single output, we designed a V-shaped composite electrode with a movable pin switch (Figure 5c). By moving the pin electrode to control the conduction and disconnection of the pathway, the hang-up mixing and directional transport of droplets can be realized (Supplementary Movie S5). Figure 5d shows the controlled mixing and transport of different colored droplets. Initially, the pin electrode is disconnected from the V-shaped pathway. Parallel droplet flows are transported along the V-shaped pathway and are hung and mixed at the bottom intersection. After mixing, the mixed droplets slide downward by shifting the pin electrode upward to make it conductive to the V-shaped electrode. By cycling the above operation, the temporal mixing of continuous droplet flows can be realized.

Benefiting from the movability of the non-contact electrodes and the fast reconfiguration of the charge-orbit patterns, ROEW realizes the dynamic handling of droplet motion by controlling the electrode switches on demand. It provides a new avenue for dynamic decision-making of droplets in reconfigurable microfluidics and has promising applications in the fields of bio-detection and chemical analysis.

## 4. Conclusions

In summary, inspired by pitcher plants and orbital Lego, we present a new reconfigurable orbital electrowetting (ROEW) technique. ROEW combines the wettability modulation mechanism of non-contact charge patterns with flexible electrodes to enable dynamically reconfigurable manipulation of droplets on inclined slippery liquid-infused porous surfaces (SLIPS). ROEW prints charge-orbit patterns on SLIPS in real-time by non-contact projection, creating precise and stable wetting pathways for continuous droplet transport. The low adhesion of SLIPS enables fast (~75 mm/s) and residue-free droplet transport. The unique features of ROEW are the fast erase-writability of charge-orbit patterns and the reversible deformation and non-contact movability of flexible electrodes. Benefiting from these features, ROEW supports flexible switching of various complex pathways on the same SLIPS, including straight lines, 90° sharp turns, and S-shaped double bends, by cyclically deforming a single reusable electrode shape. Significantly, by constructing composite electrodes with integrated switches, ROEW enables dynamic handling of droplets, such as online sorting and temporal mixing. ROEW provides a new avenue for reconfigurable microfluidics with reusable and array-free electrodes and has promising applications in reconfigurable fluidic network construction and intelligent droplet handling.

## Data Availability

The data that support the findings of this study are available from the corresponding author upon reasonable request.

## Supplementary Materials

The following supporting information can be downloaded at https://www.mdpi.com/article/10.3390/mi16060618/s1, Figure S1: Photos of experimental setup; Figure S2: Preparation of microneedle electrode arrays; Figure S3: Preparation of underlying bladed electrodes; Figure S4: Design of the underlying copper foil electrode fixing device; Figure S5: Difference in contact angle of droplets in the direction of motion; Figure S6: Controllable sliding of different kinds of droplets along a straight pathway by ROEW; Movie S1: Droplet sliding in the natural case and with charge-orbit patterns; Movie S2: Controllable transport of different kinds of droplets on inclined slippery surfaces via reconfigurable orbital electrowetting; Movie S3: Continuous transport of droplets in various pathways enabled by programming of reusable electrode shapes; Movie S4: Dynamic handling of droplet sorting enabled by controlling a composite electrode with a Y-shaped fork switch; Movie S5: Dynamic handling of droplet mixing enabled by controlling a composite electrode with a movable pin switch.
