# Supplementary material for: Reconfigurable Orbital Electrowetting for Controllable Droplet Transport on Slippery Surfaces
Source: Micromachines (Basel). 2025 May 25;16(6):618. doi: 10.3390/mi16060618 (PMC12195050; doi:10.3390/mi16060618)
Supplement: Supplementary file 1 [file micromachines-16-00618-s001.zip › micromachines-3639748-figures.pdf]

micromachines

MDPI

Supplementary Materials

Reconfigurable Orbital Electrowetting for Controllable Droplet Transport on Slippery Surfaces

Jiayao Wu [1], Huafei Li [2], Yifan Zhou [2], Ge Gao [2], Teng Zhou [3], Ziyu Wang [1,*] and Huai Zheng [1,*]

1 The Institute of Technological Sciences, Wuhan University, Wuhan 430072, China
2 School of Power and Mechanical Engineering, Wuhan University, Wuhan 430072, China
3 Mechanical and Electrical Engineering College, Hainan University, Haikou 570228, Hainan
* Correspondence: zywang@whu.edu.cn (Z.W.); huai_zheng@whu.edu.cn (H.Z.)

1. Photos of experimental setup.

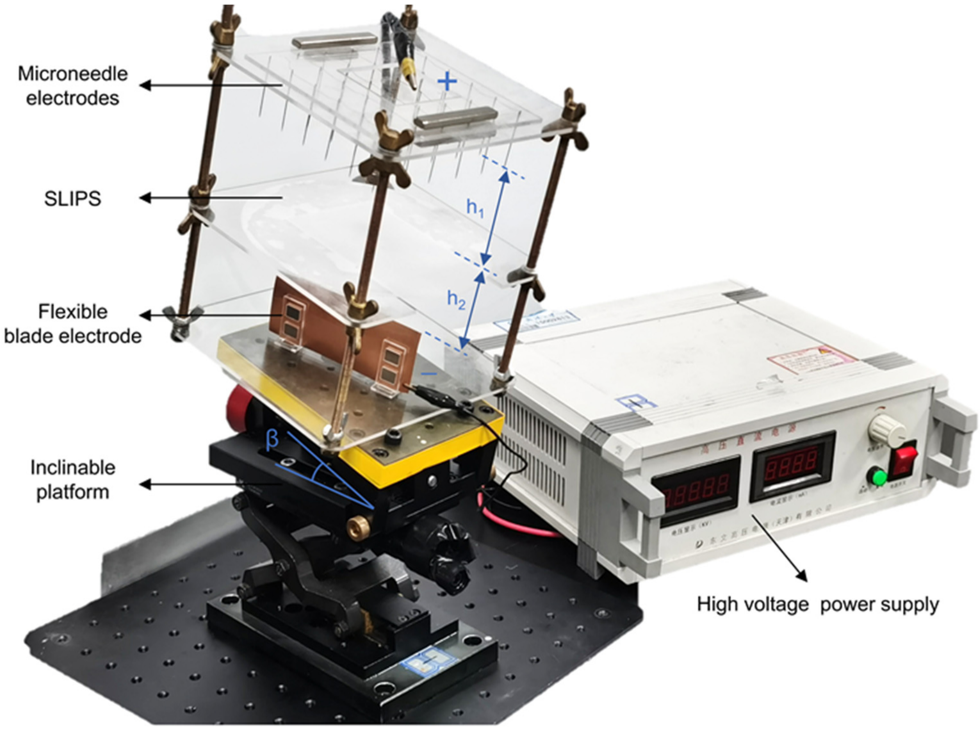

Figure S1. Photos of experimental setup. The self-built experimental setup consists of microneedle electrodes, SLIPS, flexible bladed electrode, inclinable platform, and high voltage power supply.

2. Preparation of microneedle electrode arrays.

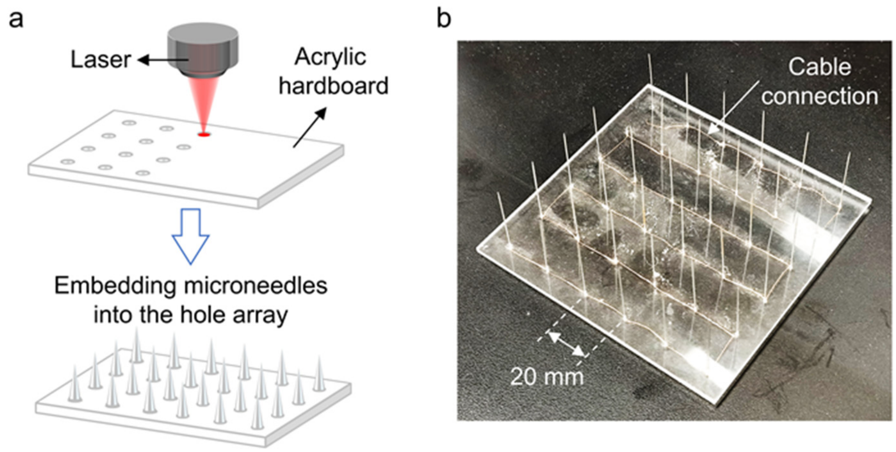

Figure S2. Preparation of microneedle electrode arrays. (a) Preparation method. (b) Effect photo.

3. Preparation of underlying bladed electrodes.

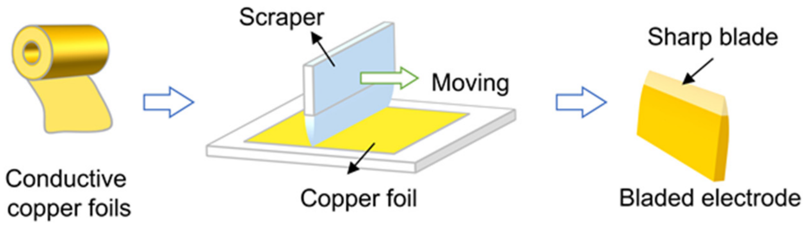

Figure S3. Preparation of underlying bladed electrodes.

4. Design of the underlying copper foil electrode fixing device.

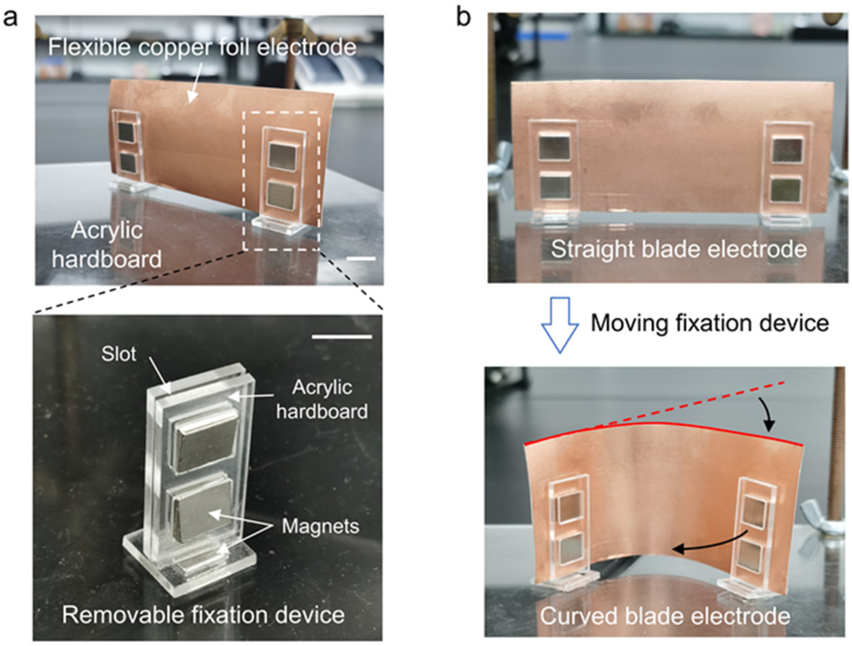

Figure S4. Design of the underlying copper foil electrode fixing device. (a) Photo of underlying copper foil electrode fixation effect. The inset shows the structural design of the removable fixation device. (b) The bending deformation of the underlying electrode from straight to curved is realized by moving the fixation device. The scale bar is 10 mm.

5. Difference in contact angle of droplets in the direction of motion.

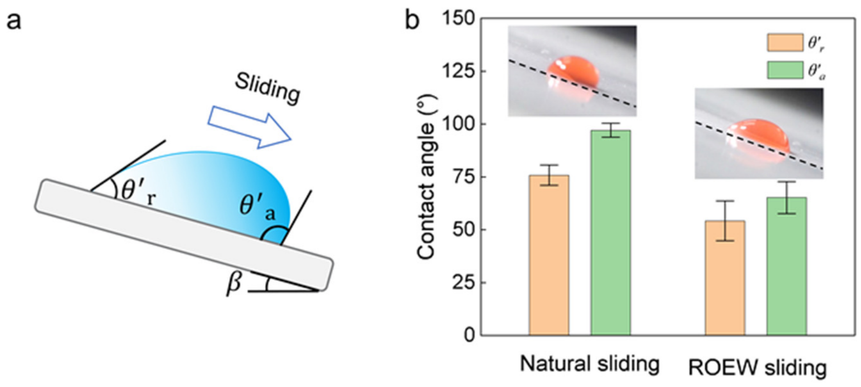

Figure S5. Difference in contact angle of droplets in the direction of motion. (a) Schematic diagram of the contact angle of a droplet as it slides down an inclined slippery surface. The contact angles in advance and rear of the droplet in the direction of motion are θ' a and θ'r , respectively. (b) Difference in contact angle in the direction of motion between droplet natural sliding and ROEW manipulated sliding. The results show that the contact angle in advance of the droplet is larger than that at the rear of the droplet for both cases of sliding. While ROEW can reduce both contact angles of advance and rear of the droplet due to electrostatic force, compared with the natural sliding of the droplet.

6. Controllable sliding of different kinds of droplets along a straight pathway by ROEW.

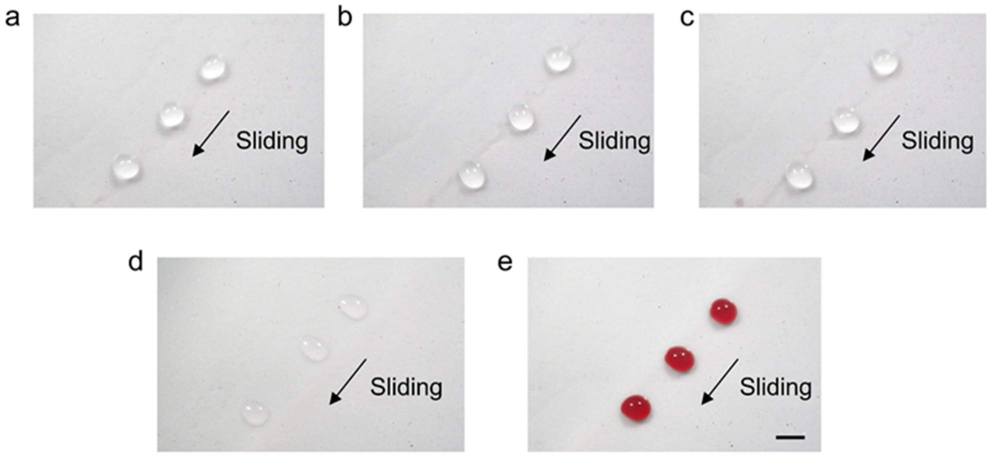

Figure S6. Controllable sliding of different kinds of droplets along a straight pathway by ROEW. (a) HCl solution. (b) NaCl solution. (c) NaOH solution. (d) Ethanol with low surface tension. (e) Colorant with high viscosity. The scale bar is 3 mm.
